# Predicting ablation zones with multislice volumetric 2-D magnetic resonance thermal imaging

**DOI:** 10.1080/02656736.2021.1936215

**Published:** 2021

**Authors:** Zahabiya Campwala, Benjamin Szewczyk, Teresa Maietta, Rachel Trowbridge, Matthew Tarasek, Chitresh Bhushan, Eric Fiveland, Goutam Ghoshal, Tamas Heffter, Katie Gandomi, Paulo Alberto Carvalho, Christopher Nycz, Erin Jeannotte, Michael Staudt, Julia Nalwalk, Abigail Hellman, Zhanyue Zhao, E. Clif Burdette, Gregory Fischer, Desmond Yeo, Julie G. Pilitsis

**Affiliations:** a Department of Neuroscience and Experimental Therapeutics, Albany Medical Center, Albany, NY, USA; b Department of Neurosurgery, Albany Medical Center, Albany, NY, USA; c Robotics Engineering Department, Worcester Polytechnic Institute, Worcester, MA, USA; d GE Global Research Center, Niskayuna, NY, USA; e Acoustic MedSystems, Inc, Savoy, USA; f Animal Resources Facility, Albany Medical Center, Albany, NY, USA

**Keywords:** Brain metastases, focused ultrasound, magnetic resonance-guided robotically assisted delivery, needle-based therapeutic ultrasound, magnetic resonance thermal imaging

## Abstract

**Background:**

High-intensity focused ultrasound (HIFU) serves as a noninvasive stereotactic system for the ablation of brain metastases; however, treatments are limited to simple geometries and energy delivery is limited by the high acoustic attenuation of the calvarium. Minimally-invasive magnetic resonance-guided robotically-assisted (MRgRA) needle-based therapeutic ultrasound (NBTU) using multislice volumetric 2-D magnetic resonance thermal imaging (MRTI) overcomes these limitations and has potential to produce less collateral tissue damage than current methods.

**Objective:**

To correlate multislice volumetric 2-D MRTI volumes with histologically confirmed regions of tissue damage in MRgRA NBTU.

**Methods:**

Seven swine underwent a total of 8 frontal MRgRA NBTU lesions. MRTI ablation volumes were compared to histologic tissue damage on brain sections stained with 2,3,5-triphenyltetrazolium chloride (TTC). Bland-Altman analyses and correlation trends were used to compare MRTI and TTC ablation volumes.

**Results:**

Data from the initial and third swine’s ablations were excluded due to sub-optimal tissue staining. For the remaining ablations (*n* = 6), the limits of agreement between the MRTI and histologic volumes ranged from −0.149 cm^3^ to 0.252 cm^3^ with a mean difference of 0.052 ±0.042 cm^3^ (11.1%). There was a high correlation between the MRTI and histology volumes (*r*^2^ = 0.831) with a strong linear relationship (*r* = 0.868).

**Conclusion:**

We used a volumetric MRTI technique to accurately track thermal changes during MRgRA NBTU in preparation for human trials. Improved volumetric coverage with MRTI enhanced our delivery of therapy and has far-reaching implications for focused ultrasound in the broader clinical setting.

## Introduction

Surgical resection with concurrent biopsy and subsequent whole brain radiation therapy (WBRT) have become the mainstay of treatment for brain metastases. With current treatment options, 8.1% of brain metastases patients survive on average for 2 years, 4.8% for 3 years, and 2.4% for 5 years [1]. Common complications for WBRT may include leukoence-phalopathy, brain atrophy, and learning and memory impairments, leading to a preference for stereotactic radiosurgery (SRS) when possible [2,3]. However, both WBRT and SRS increase the risk of radiation toxicity [4]. Thermal therapies including transcranial magnetic resonance (MR)-guided focused ultrasound (tcMRgFUS) and laser interstitial thermal therapy (LITT) are being explored as alternative treatment options [5-7].

Our group has proposed delivery of needle-based therapeutic ultrasound (NBTU) as an alternative strategy as it produces less collateral damage to critical structures such blood vessels and can target lesions adjacent to the skull-base and calvarium [6,8,9]. Further, we deliver the therapy using a magnetic resonance-guided robotically-assisted (MRgRA) platform [6,10]. Previously, we developed the tools to test the feasibility of this novel neuroablative technique in survival swine [10]. However, one issue for our therapy, which is also experienced in other thermal therapies, is the reliance on single-slice 2-D MR thermal imaging (MRTI), commonly employed as a means of tracking ablation-induced temperature changes. MRTI strategies which rely on a single brain slice centered at the treatment focal zone [11] and repetitive 2-D sequences in multiple planes with incomplete volumetric reconstructions [12] do not have the precision to test the limits of these therapies; specifically, there is delayed thermal feedback and practical magnitude limitations [13].

Here we employ a contiguous multislice 2-D (volumetric) MRTI technique that measures temperature variations in realtime. The multiple slices create volumetric reconstructions of the targeted brain region and minimize undetected tissue between slices [13]. This study evaluated the correlation between the MRTI-predicted ablation zones and observable tissue damage from MRgRA delivery of NBTU in a swine model by comparing ablation volumes from intraoperative volumetric MRTI thermal dose maps and postmortem histologic sections stained with 2,3,5-Triphenyltetrazolium chloride (TTC).

## Methods

### *In-vivo* swine model

This project was approved by the Albany Medical College (AMC) Institutional Animal Care and Use Committee (IACUC). MRgRA delivery of NBTU was conducted on male and female *Sus scrofa domesticus* swine (8–20 weeks old, 10 weeks on average) weighing between 18 kg and 25 kg. Four acute swine (sacrificed immediately post-procedure) and three subacute swine (sacrificed 1–4 days post-procedure) were employed to create a total of 8 lesions.

### NBTU applicators to induce tissue hyperthermia

The NBTU applicators have been designed to allow for multiple, cylindrical piezoelectric sectored transducers aligned along the longitudinal axis of the probe [6,14]. Each transducer was 7 mm (mm) in length with a 1.5 mm outer diameter and could be independently activated to emit a radial 180° or 360° insonation pattern within the target tissue. The transducers with a 180° ablation pattern were specially sectioned to allow acoustic energy to be transmitted directionally. The exact insonation pattern may be influenced slightly by the eccentricity of the cylindrical transducers during the manufacturing process, tissue heterogenicity, and local perfusion [6,15]. The applicators were mounted in a Celcon catheter which was designed to allow degassed water circulation through the casing around the applicator and the transducers at 20 mL/minute to prevent transducer overheating, provide acoustic coupling, increase energy delivery, and decrease treatment times [6]. For these experiments, NBTU applicators with a single element were used with a continuous waveform with an operating frequency ranging from 6.8 to 8.5 MHz and acoustic efficiency ranging from 55 to 75%. Both the acoustic power and duration of dose delivery for this study were pre-selected based on our previously acquired swine data [10] in which 3–6 W had been used from 100 to 180 s. The user entered the acoustic power and duration and during none of the experiments were the sonications manually ended. A closed loop system, varying the probe direction or treatment duration based on a certain themal dose threshold, is being investigated and will be incorporated in future iterations of our software.

### MRgRA delivery of NBTU – Workflow

For each subject, general anesthesia was induced with a combination of injectable anesthetics. Powdered tiletamine and zolazepam (100 mg/mL) was reconstituted using 2.5 mL ketamine (100 mg/mL) and 2.5 mL xylazine, and administered intramuscularly. The subject was then intubated and maintained with isoflurane at 1–5% in 1 L/min of oxygen. The scalp was then prepped and draped in sterile fashion. A paramedian incision was marked and the area was infiltrated with 5 mL of bupivacaine prior to incision. A #10 blade was used to make an incision and bovie electrocautery was used to dissect down to the calvarium. A Penfield #1 was used to elevate the periosteum from the calvarium, and self-retaining retractors were placed. A Midas Rex high-speed drill (Medtronic, Dublin, Ireland) was used to create roughly 1 cm right, left, or bilateral frontal burr holes (Table 1). The edges of the burr hole were undercut using a #2 Kerrison. The dura was then opened using a #11 blade in a cruciate fashion, and the dural edges were coagulated using bovie electrocautery. The pia was coagulated using bovie electrocautery followed by a small corticotomy using the #11 blade. The wound was then irrigated using sterile saline and small piece of Surgifoam (Ethicon, Somerville, NJ, USA) was placed in the burr holes to minimize cerebrospinal fluid egress. The incision was then temporarily closed with Dermalon sutures in a serial interrupted fashion.

**Figure 1.**
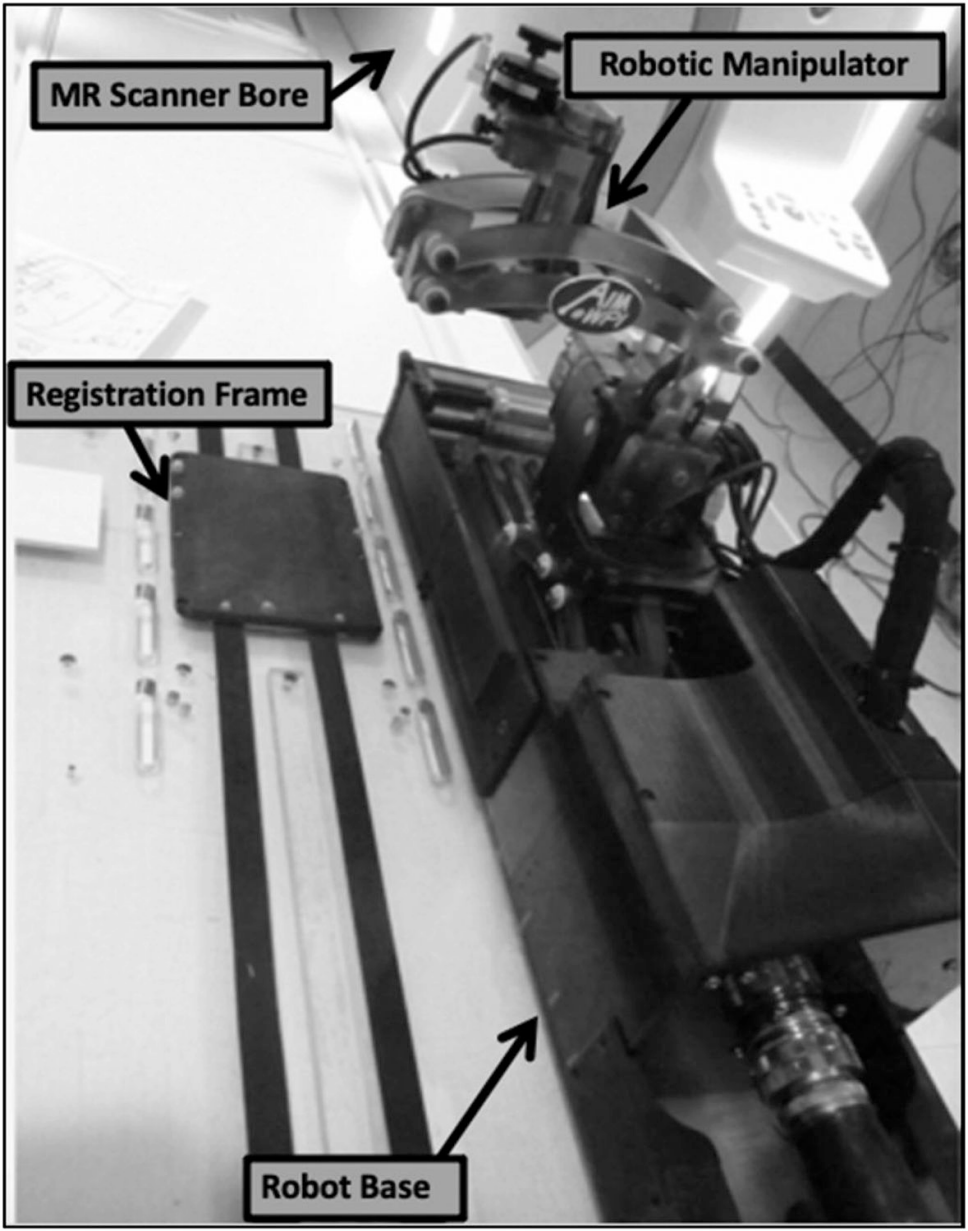
Robotic manipulator positioned near the MR scanner.

The swine were then transported to the MR scanner and placed in a prone position with the burr holes aligned with the robot arm to allow target access. The robot was physically locked onto the MR scanner rails and was registered to the MR scanner’s coordinate system *via* a registration frame located at the base of the robot (Figure 1). The MR-compatible 7-degree of freedom (DOF) robot mirrors the kinematics of a Leksell stereotactic frame (Elekta AB, Stockholm, Sweden) and utilizes 5-DOF for NBTU applicator positioning and orientation and 2-DOF for insertion and rotation [6].

3D T1 BRAVO MR images (TE 3.3, TR 8500, 1.6 mm slice thickness) were acquired to determine the cannula entry point to the cranial vault and the data was transferred to the TheraVision software (Acoustic MedSystems, Inc., Savoy, IL, USA) that controls the robot. The incision was re-opened, and the robot was aligned to insert the cannula to a programmed planned depth. The robot then inserted the MR-compatible ACOUSTx^®^ NBTU applicator (Acoustic MedSystems, Inc., Savoy, IL, USA) through the cannula to the target location.

**Figure 2.**
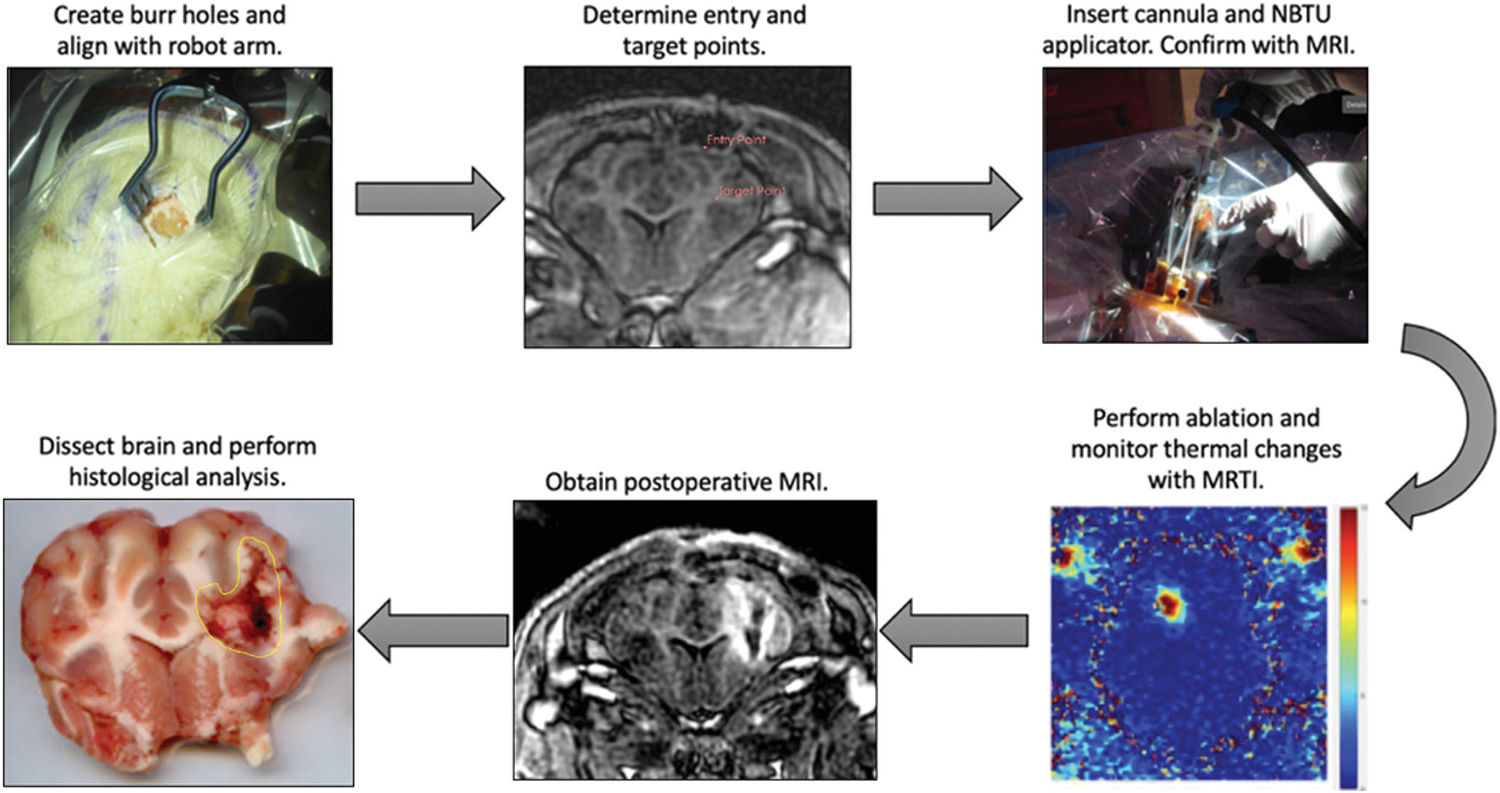
Clinical workflow of MRgRA delivery of NBTU. Top, left – a burr hole craniotomy is performed to gain access to the intra-cranial space. Top, middle – the Entry Point and Target Point are selected using our TheraVision software (Acoustic MedSystems, Inc., Savoy, IL, USA) and the coordinates are sent to the robot. Top, right – the robot moves into position and the cannula and ACOUSTx^®^ NBTU applicator (Acoustic MedSystems, Inc., Savoy, IL, USA) are mounted to the robot. The robot then inserts the probe to target depth. Bottom, right – the ablation ensues and thermal changes are monitored using MRTI. Bottom, middle – post operative MRI images are obtained. Bottom, left – the brain is dissected and histologic analysis is performed using TTC staining. The area of damage is calculated using ImageJ (NIH funded open access software).

Multiple 3 or 5 mm spaced slices were then overlay on the intraoperative MR images orthogonal to the ablation probe as ROIs to create a multiplanar thermal dose map with which the volumetric MRTI algorithm was used to track thermal dose changes. After selecting these regions, we performed a low dose test ablation at 1.5 W for 30 s to verify the shape and direction of the ablation volume prior to commencing with full treatment. Once the ablation was completed, post-ablation T1, T2, diffusion-weighted (DWI), and apparent diffusion coefficient (ADC) MR images were acquired and the applicator and cannula were removed. The acute swine were euthanized immediately post-procedure with an intravenous injection of pentobarbital sodium with phenytoin, under general anesthesia, while the subacute swine were euthanized 1–4 days post-procedure. The brains were dissected from the subject immediately following euthanasia and hardened in a −80 °C freezer or on dry ice for approximately one hour in preparation for TTC staining. The workflow is demonstrated in Figure 2.

### MRTI thermal dose maps

MRTI was achieved using a fast single-echo spoiled gradient echo (SPGR) imaging sequence to calculate referenced phase difference maps for use in a corrected proton resonance frequency shift (PRFS) technique. Our group also developed a referenceless MRTI technique (using the Goldstein algorithm [16] for phase unwrapping and a 4th order spatial polynomial fit to the unwrapped phase in the non-heated outer edge as the background phase for subtraction) to employ in the event that the swine had significant motion; however this was never experienced during the experiments, and only the referenced method was used for data acquisition. We note that our choice of a fast SPGR multislice 2D MRTI protocol has previously been optimized and down-selected from a number of stock MR sequence options. This approach has been shown to provide maximum temperature measurement precision and volume coverage in the presence of image artifacts arising from the NBTU applicator operation *in vivo* [17]. The sequence parameters included echo time (TE) = 13.2 ms, repetition time (TR) = 93 ms, flip angle (theta) = 30, field of view (FoV) = (15 × 15)cm^2^, matrix size = (256 × 128, zero filled to 256 × 256), 5 slice acquisition, and slice thickness = 3–5mm. Corrected PRFS maps are computed as:

(1) $$\Delta T(x,y)=\frac{\Delta \phi_{w}(x,y)-\Delta \phi_{cor}(x,y)}{\gamma \alpha B_{0}TE}$$

where ɸ *_cor_(x,y)* represents the image phase in a non-heated region of the brain, assumed to be dominated by static magnetic field (B0) drift. Δɸ *_w_(x,y)* represents the water based phase difference maps, which are corrected by the interpolated phase difference maps of the Δɸ*_cor_(x,y)*.

Here the referenced PRFS-MRTI maps were corrected by 2nd order spatial polynomial fit to phase difference maps of the non-heated outer edge region of the swine brain [18]. In the referenced PRFS MRTI method, a 2nd order polynomial correction was used. Based on our experience, a 2nd order performed best when compared to a 1st or 3rd order polynomial in minimizing the temperature standard deviation in the brain region during baseline scanning. The correction regions of non-heated brain tissue were manually drawn prior to MRTI data acquisition. These regions were drawn on each MRTI plane as a 3-pixel-width contour around the outer edge of the visible brain. This region was selected as a reference because it minimized susceptibility from the probe, surrounding instruments, air, and tissue interfaces. For these experiments, the PRFS thermal coefficient for water-based tissues (α) was not experimentally calibrated [19]. Hence, a nominal value of a = −0.01 ppm/°C was chosen.

In order to create a volumetric map of the temperature gradients for each sonication, the maximum temperature intensities at varying distances from the region of interest (ROI) were calculated in 3 or 5 mm thick slices [20]. Temperature measurements can depict the absolute temperature changes or relative temperature changes compared to un-heated tissue or to reference data [21]. The thermal damage is traditionally quantified using the Sapareto-Dewey equation, and the cumulative equivalent minute (CEM) standard delineates the boundaries of the ablation zone [22,23]. The CEM43 is defined as the equivalent time the target tissue temperature has to be at 43 °C to induce thermal damage [22]. We selected a CEM43 of 70 based on our past experience with ablation in liver tissue [24-26] as well as a comparison to similar literature [27] and studies demonstrating a CEM43 of 50 – 240 range to induce necrosis in soft tissue [28]. Employing the formulation of Sapareto and Dewey, the thermal dose calculated from the temperature from MRTI image is given by:

(2) CME43=∑t=0t=finalR(43−Tt)Δt,{R=0.25forT<43°CR=0.50forT≥43°C}

where *T_t_* is the average temperature during time Δ*t*. The unit of thermal dose is equivalent minutes at 43° C.

**Figure 3.**
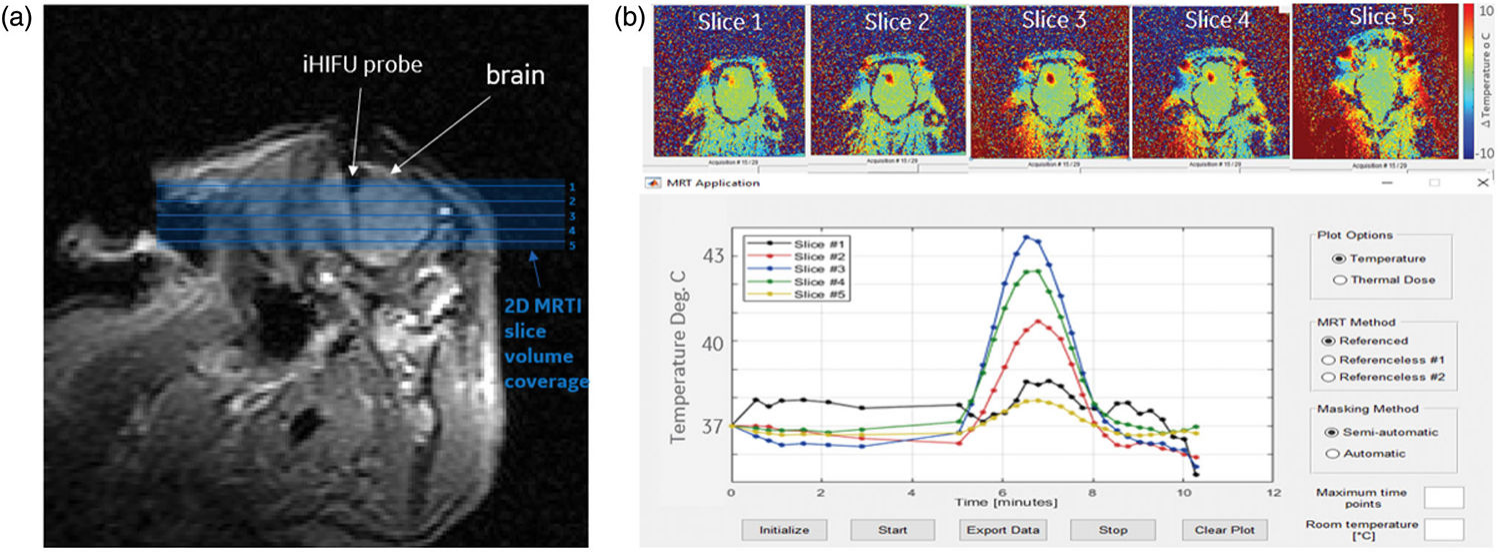
MRTI temperature maps from one acute swine (a) MRI image showing probe location after insertion into swine’s brain. Orthogonal, contiguous MRTI slice placement is shown with shaded blue background indicating the volume of tissue being imaged. (b, top) MRTI maps for slices labeled in (a) set to the maximum heating timepoint. (b, bottom) Improved MRTI software user interface with real-time average temperature plot for a user-drawn ROI in MRTI map.

The MRTI system measured the temperature changes in multiple slices simultaneously to create a volumetric reconstruction of the thermally damaged zone. For these experiments, five contiguous slices that covered the probe insertion ROI were simultaneously visualized within 10 s (Figure 3(a,b), top). The MRTI software measured the 2-D real-time average temperature and distribution for a user-defined ROI in each of the slices to create a plot of temperature versus time (Figure 3(b), bottom). Volumetric thermal dose (CEM43) maps were also created.

**Figure 4.**
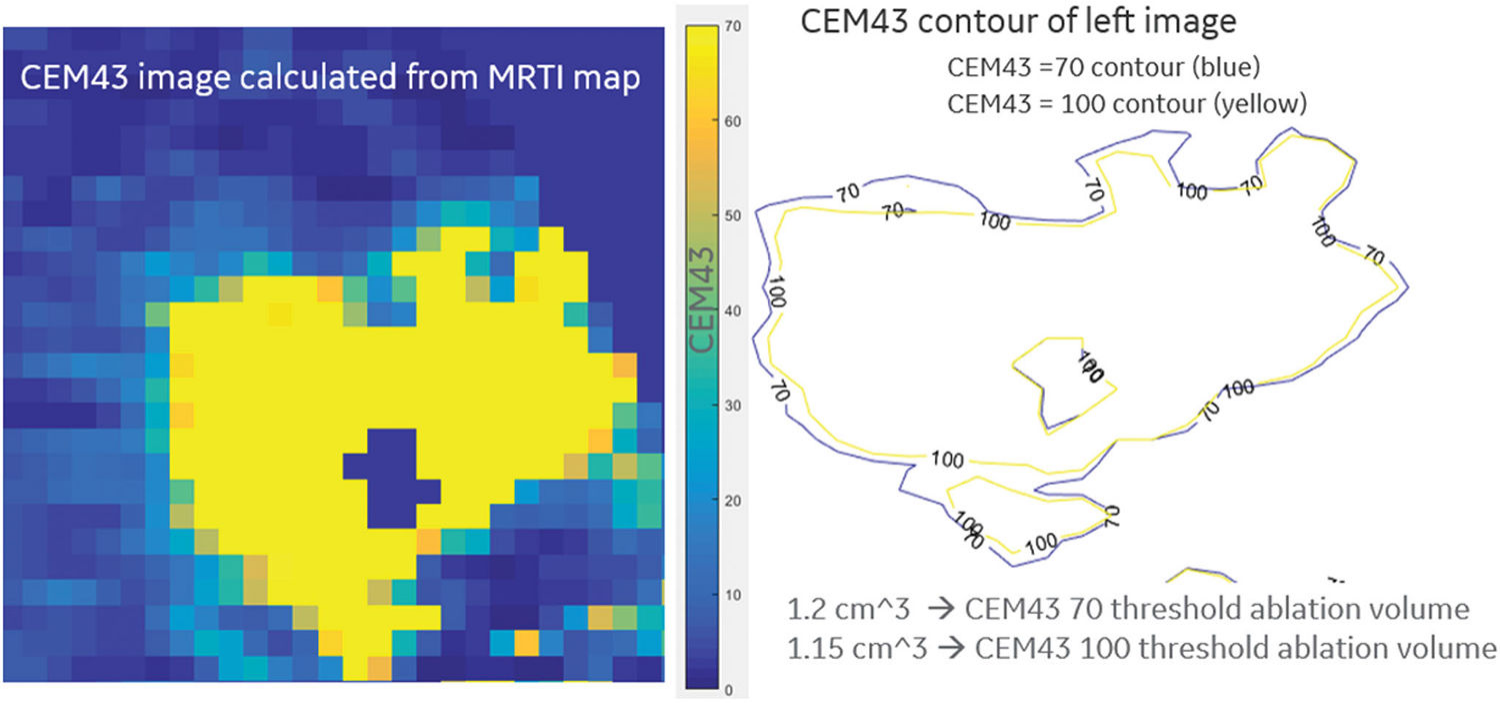
CEM43 dose maps. Left – Pixel grid of CEM43 dose delivered via NBTU. Right – CEM43 isodose lines derived from the pixel grid. The volume of the necrotic zones was calculated by counting the pixels at the specified CEM43 threshold and multiplying by the 3 pixel dimensions.

The volumetric MRTI measured the temperature changes at different distances from the focal point, and the necrotic zone boundaries were measured at CEM43 > 70. The volume of the necrotic zones was calculated by counting the pixels at the specified CEM43 threshold and multiplying by the 3 pixel dimensions. This is demonstrated in Figure 4.

### Histological analysis

TTC staining (2% weight/volume) was employed to examine ablation size. The dissected swine brain was hardened in a −80 °C freezer or on dry ice for one hour. The brain was aligned with the Zivic Pig Brain Slicer (Zivic Instruments, Pittsburgh, Pennsylvania) and 5 mm slices were made from the frontal to occipital poles. Each slice was placed in a culture dish containing phosphate-buffered saline with azide (PBS). The PBS was replaced with enough TTC solution to cover the slices. Each slice was incubated in a 37 °C incubator for 4–15 min until the slices turned light pink. Each slice was flipped and re-incubated for 4–15 min until the slices turned dark pink. The TTC solution was removed and each slice was washed with PBS before paraformaldehyde (PFA) was added to fix the tissue. The slices were photographed. After the slices were incubated with PFA for 24 h in 4 °C, the PFA was replaced with PBS [29]. The viable tissue was stained and the damaged tissue remained unstained [29].

### Ablation volume calculations

**Figure 5.**
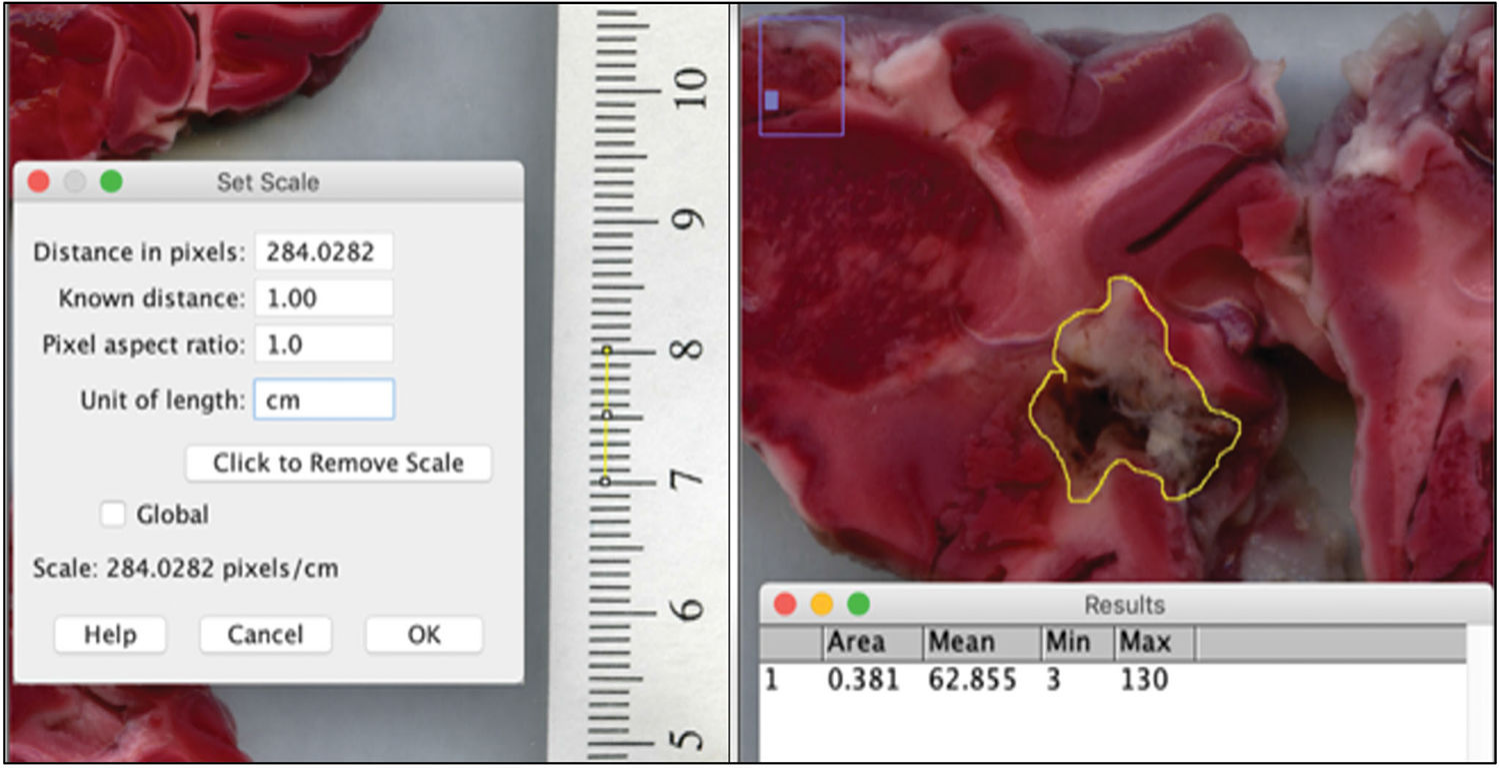
The ablation volume is calculated using ImageJ (NIH funded open access software). Left – a ruler adjacent to the coronal brain sections is used to calibrate the software between pixels and cm. Right – the area of ablation damage is outlined and the software calculates the circled area.

Pictures of the slice images were opened on ImageJ (NIH funded open access software) [30], and a line spanning 1 cm on the imaged ruler was used to set the scale (Figure 3, left). An area was drawn around the margins of the ablated area with the lasso feature and the program calculated the area of the circled region in cm^2^ (Figure 3, right). Each ablated area was multiplied by the 5 mm slice thickness if the ablation went through the posterior side of the slice. The ablation volumes for all of the slices exhibiting ablation were added to get the total ablation volume (cm^3^). Two individuals labeled P1 and P2 independently repeated the ablation volume measurements to account for observer bias (Figure 5).

### Statistical analysis

MRTI and histologic volumes were compared using Bland-Altman analysis with the limits of agreement defined as the mean difference ± 1.96 standard deviations of differences [31], linear regression models with the ideal correlation set at R^2^ > 0.8, and the paired student *t*-test. Absolute maximal tissue temperatures and times for temperatures to return to baseline were reported as means ± standard errors (SEM). The student *t*-test was used to determine significance at *p* < .05 using GraphPad Prism software (La Jolla, California, USA).

## Results

Seven swine (5 male, 2 female) underwent a total of 8 frontal MRgRA NBTU lesions. Four swine were euthanized immediately following the procedure and 3 were observed for 24–72 h. The average age was 10.2 weeks and average weight was 20.92 kg. The ablation doses and duration are listed in Table 1. Data from the initial and third swine’s ablations were excluded due to sub-optimal tissue staining. In the initial swine ablation, the tissue was overstrained turning the tissue deep red and making it difficult to differentiate between the normal and damaged tissue. Likewise, in the third swine ablation, the tissue was under-stained, with most tissue remaining pale and leading to difficultly in contrasting normal tissue from damaged tissue. Thus, both of these volumes were overestimations because the actual margins of damaged tissue were poorly differentiated. For the remaining ablations (*n* = 6), the mean ± SEM absolute maximal temperature increase during the ablation was 13.58 ± 2.20 °C. The mean ± SEM time for the temperature to return to baseline was 122.86 ± 12.09 s.

MRTI and histologic volumes were compared to correlate regions of predicted and actual damage (Table 1). The limits of agreement between the MRTI and histology ablation volume measurements ranged from −0.149 cm^3^ to 0.252 cm^3^ with a mean difference of 0.052 ±0.042 cm^3^ (11.1%). There was a high correlation between the MRTI and histology volumes (*r*^2^ = 0.831) with a strong linear relationship (*r* = 0.868). There was no significant difference between the predicted MRTI volumes and measured histologic volumes (*p* = .2708). There was a stronger correlation between the histology and MRTI-predicted ablation volumes (*r*^2^ = 0.831) in this study compared to our previous work (*r*^2^ = 0.138) [10].

### Observer variability

The limits of agreement between the MRTI-predicted volumes and histologic ablation volumes measured by P1 ranged from −0.220 cm^3^ to 0.313 cm^3^ with a mean ± SEM difference of 0.047 ± 0.056 cm^3^. The limits of agreement between the MRTI-predicted volumes and histologic ablation volumes measured by P2 ranged from −0.195 cm^3^ to 0.305 cm^3^ with a mean ± SEM difference of 0.055 ± 0.052 cm^3^. There were no significant differences in the ablation volumes measured between P1 and P2 (*p* = .957).

## Discussion

In this study, we successfully performed 8 MRgRA NBTU lesions in 4 acute swine and 3 subacute swine while monitoring the thermal ablation using volumetric 2-D MRTI. The multislice MRTI system predicted the volumetric ablation zones seen in postmortem histological sections at an R^2^ > 0.8 and better predicted the actual tissue damage compared to the previous single slice 2-D MRTI system. Our findings demonstrated a low percent difference between the MRTI and histologic data (11.1%) which was comparable to similar studies in the literature [27].

### MRTI advancement over previous implementation

In contrast to the previous implementation of a single-slice 2-D MRTI technique [10], the current multislice MRTI created proper volumetric thermal dose calculations to measure thermal doses in the targeted region and the surrounding tissue within seconds. In the single-slice thermometry, excitation and analysis of each slice occurred sequentially, and repeated sonications in different imaging planes were needed to approximate a single volumetric reconstruction [11,13]. At least three sonications were needed to identify the heating focal zone, leading to time delays and therefore incomplete information [11]. In contrast, volumetric MRTI increased the speed, resolution, and accuracy of the thermal dose maps and volumetric reconstructions [13]. Multislice thermometry shortened treatment times by verifying the heating focal points with a single sonication [11]. The new multislice MRTI algorithm can acquire real-time temperature maps for 5 slices simultaneously in 10 s, providing enough time to alter ablation parameters to minimize heating outside the targeted region.

The previously utilized single-slice MRTI technique did not correct for external magnetic field drifts, decreasing the accuracy of temperature readings and ultimately underestimating the delivered thermal dose [21]. Our current multislice MRTI technique corrects for background magnetic field drift by using a spatial polynomial fit to phase difference maps of a non-heated region of the swine brain. The phase correction ensures that any reported phase changes are induced by real temperature changes instead of field drifts. Previous heated phantom experiments have demonstrated that the drift correction technique had high resolution and drift-free stability [32]. We similarly demonstrated enhanced resolution and accuracy of temperature readings using our corrected MRTI technique compared to the previous non-corrected technique in a swine model.

### Use of directional NBTU

The NBTU clinical workflow is very similar to the procedure used to deliver LITT, however NBTU has the advantage of being able to provide directional heating patterns [10]. Early *in vivo* attempts at NBTU in a canine model demonstrated the feasibility of a minimally invasive, trans-urethral directional ablation of prostate tissue in a 180° pattern; MRTI was also used to monitor the ablation during these experiments [33]. Recent large animal studies have demonstrated the feasibility of NBTU in combination with chemotherapy for the treatment of liver cancer [24]. Burtnyk *et al* have similarly used directional ultrasonic energy for the creation of thermal ablative lesions in a porcine brain model [27]. Their model uses a closed loop system with collimation for creation of conformal lesioning. Our probes rather use sectored piezoelectric transducers for generation of conformal patterns. We have previously demonstrated the use of MRgRA NBTU in a survival swine model to create MRTI-monitored intracranial ablative lesions [10]. The MR-compatible ACOUSTx^®^ NBTU applicators (Acoustic MedSystems, Inc., Savoy, IL, USA) are able to perform ablations of various angles including 360° and 180°, and also has narrow-beam and very-narrow-beam angular insonation configurations (90°, 60°) [15]. The minimally invasive catheter design and ability to achieve directional conformal ablations make NBTU an attractive tool for stereotaxy. In this study we were able to create 180° lesions as well as circumferential ablations. The implementation of a closed loop system for additional conformal patterns is currently under investigation and will be used in future iterations of the system.

NBTU applicators employ longitudinally stacked, sectored piezoelectric transducers to deliver focused ultrasonic (FUS) energy on a target region, thereby increasing tissue temperature and leading to protein denaturation, DNA breakdown, and coagulative necrosis [34]. While tcMRgFUS similarly uses FUS energy, the system remains extracranial, limiting the geometric patterns to small, ovoid focal zones and greatly attenuating the transmitted energy as it passes through the calvarium, leading to prolonged treatment times [7,35]. In addition, tissue diagnosis is not possible and the system is not able to target lesions near the skull base or calvarium [36]. LITT creates hyperthermia and tissue damage *via* the absorption of high-intensity light and is also currently in use as a stereotactic ablative therapy, including the NeuroBlate system (Monteris Medical Corp., Plymouth, Minnesota, USA) and Visualase system (Medtronic, plc., Dublin, Ireland); however, due to the high temperatures achieved by these systems they cannot be used safely for vascular lesions or areas adjacent to critical vasculature [5,37]. Conversely, FUS has been shown to be preferentially less damaging to blood vessels [8].

### Use of MRTI technology in other thermal therapies

A hurdle to the clinical application of thermometric ablative therapies has been the control of thermal dose applied to the tissue and limitation of collateral damage to the surrounding non-targeted regions. Advances in MRTI have greatly helped in this endeavor. In 1995, DePoorter described a linear relationship between proton resonance frequency and temperature as described by the screening and volume susceptibility constants [38], heralding the use of phase-mapping as a way of tracking thermal dose [21]. Since that time, MRTI has been investigated for the monitoring of hyperthermic lesioning methods such as LITT and tcMRgFUS.

In 2004, the feasibility of MRTI as a feedback control system for LITT was demonstrated in 12 *ex vivo* and 16 *in vivo* lesions in a canine model; the authors concluded that this approach aided in regulating heating, eliminating carbonization and vaporization, and protecting the fiberoptic applicators [39]. Further attempts at correlating real-time MRTI with tissue damage and cell death in LITT were demonstrated in a rabbit model [40]. MRTI-guided LITT has also been validated as a minimally-invasive alternative to open craniotomy in four patients with recurrent glioblastomas (GBMs) with no permanent peri-operative neurologic adverse effects reported [41]. A variety of 2D and 3D MRTI protocols are being actively investigated for LITT [42].

MRTI has also become a mainstay of monitoring treatment during lesioning in tcMRgFUS. A study of 120 patients undergoing tcMRgFUS mediated thalamotomy demonstrated the ability to estimate the applied cumulative thermal dose and accurately predict lesion shape [43]. Visualization of neuro-anatomic structures using acquired MRTI data in tcMRgFUS may also have further potential to improve targeting accuracy without adding additional treatment time [44]. The use of volumetric MRTI has also recently been described with MRgFUS for the treatment of 10 patients with non-palp-able T0 stage breast cancer with good success [45]. Continued advances in MRTI have the potential to improve the safety, efficacy, and reliability of MR-guided cancer treatments.

### Limitations

The current MRTI system and imaging protocols were optimized using non-pathological swine brain tissue. Pathological and normal brain tissue vary in consistency and acoustic properties [46]. In addition, different types of brain tumors have variabilities in their vascularities and consistencies [46]. We aim to confirm the NBTU directional ablative patterns and measure heat dissipation in phantom models designed to mimic the consistency of brain tissue and softer and firmer brain metastases [46]. Additionally, the first and third swine brains were inadequately stained due to sub-optimal tissue incubation times in the TTC solutions and were excluded from the data analysis. The NBTU probe did not accurately reach the target region in the first ablation due to difficulties positioning the swine head, which were subsequently improved in later trials.

## Conclusion

In preparation for human clinical trials, we optimized a fully integrated multislice volumetric 2-D MRTI technique to accurately measure temperature changes in real time during NBTU ablations. This technique limits collateral tissue damage to the non-targeted regions and critical neurovascular structures during treatments, which provides theoretical advantages over current treatment options and may lead to improved patient outcomes.

## Figures and Tables

**Table 1.** Ablation parameters and volumes.

	Acuity	Side	Probe	Acoustic Power (W)	Duration (s)	Absolute Maximal Temperature Increase (°C)	Time to return to baseline (s)	Histology volumes (cm^3^)	MRTI volumes (cm^3^)
Swine 1	Acute	Center	360°, 7mm	3	100	N/A	N/A	N/A	N/A
		Right	360°, 7mm	3	100	13.00	135	2.49***	0.15
Swine 2	Acute	Center	360°, 7mm	3	100	22.00	100	0.25	0.30
		Right	360°, 7mm	4	100	23.07	100	0.60	0.35
Swine 3	Acute	Center	360°, 7mm	4	120	7	150	3.693***	0.4
Swine 4	Acute	Right	360°*, 7mm	6	180	16	180	0.92	0.9
Swine 5	Subacute	Center	360°, 7mm	3	120	8	120	0.44	0.4
Swine 6	Subacute	Right	360°, 7mm**	3	120	9.6	120	0.48	0.45
Swine 7	Subacute	Right	180°, 7mm	3	120	10	90	0.27	0.25

* The probe used for this ablation ablated slightly less than 360° circumferentially (about 320°); this probe was replaced for the next ablation.

** The robot stalled and the NBTU probe was manually inserted.

*** Suboptimal tissue staining led to loss of demarcation of damaged tissue.
